# A Case of Double Ovariotomy.—Operation by Professor James P. White

**Published:** 1881-07

**Authors:** C. M. Daniels


### A CASE OF DOUBLE OVARIOTOMY.—OPERATION BY PROFESSOR JAMES P. WHITE.

REPORTED BY C. M. DANIELS, M. D.

MRS. W., aged 45, menstruated first at 16, afterward regularly; married at 24; never pregnant; never suffered from any severe illness or injury. Menstruation continued normal until about Sept., 1880, then passed two months with but little show of color, and suspected possible pregnancy. Following month re-appeared natural, *and continued so up to time of operation*. Noticed abdominal enlargement about Nov. 1st, following apparent cessation of menses, together with an œdematous condition of lower limbs. Great lateral abdominal pain soon followed and increased with advancement of tumor's growth, the slightest movement causing great suffering. Came to Buffalo April 28, 1881, for consultation, etc. Ovarion tumor diagnosed, and an early operation advised. Patient for one week put upon tonic treatment, with diet as stimulating as possible. Operation May 5th; patient under ether; exploratory incision in median line half way between umbilicus and pubis, a uterine dilator passed through opening and used as a probe or "long finger" passed around a large tumor, showing only slight adhesions which separated readily. Abdominal opening enlarged to about five inches and a large trocar passed into tumor, showing but slight flows of fluid; further examination showed the contents of the several cysts (the tumor being multilocular) about twelve in number, was composed of a substance closely resembling glue. Passing antero-posteriorly through tumor, the contents of each cyst as opened seemed to be more dense and adhesive than the one before it, making the operation very difficult as the semi-solid and gluey material adhered firmly to everything with which it came in contact. The cysts being severally emptied and sack drawn through abdominal incision showed a large and very vascular pedicle, which was ligated with extra strong carbolized silk ligature within half an inch from uterus. All bleeding ceased, pedicle divided close to ligature. Examination on opposite (left) side disclosed another tumor about twice the size of a goose egg, pedicle small and easily ligated and tumor removed without difficulty; examination showed contents to be of the same peculiar semi-fluid material as the other, although of less density.

Uterus found to be of normal size, etc. The abdominal cavity being very carefully and thoroughly cleaned of all foreign material, with soft carbolized sponges, and until all oozing from divided tissues had ceased, the ligatures divided close down to knot on pedicles and returned to abdominal cavity. Prof.

White's custom is to use the actual cautery on pedicle, but strong tendency to hemorrhage in this case made ligatures necessary. External wound closed by five wire sutures, approximating the divided peritoneum and deep as well as superficial edges of the incision, equally. An equal number of superficial silk sutures being used to join integument.

A carbolized compress with five long adhesive straps one and one-half inches in width, passing entirely around body, drawn tight, completed dressing in usual manner, and patient placed in bed. Morphia in moderate quantities relieved all pain. Perfect rest to stomach enjoined; all nourishment being given per rectum for first three days. A little nausea on second day, but no vomiting. Temperature 101 to 102½, and at one time reached 103 for a few hours. Ten grains quinine given per rectum, twice daily, during highest range of temperature, being all the medication required.

There are two peculiar circumstances connected with this case, aside from its general interest, that perhaps may well merit attention, and which induced this report. On the sixth day after operation, a well-marked case of "mumps" developed on right side and passed through a very active and painful course, and doubtless caused temperature to continue higher than it otherwise would have done; this suggesting the possibility of its being a sympathetic inflammation reversely analogous to that which sometimes occurs in the male. Also, that she menstruated regularly until *one week* previous to operation, although both ovaries were the seat of the disease, the larger (right) tumor weighing about twenty-five pounds and the smaller one ten to twelve ounces. Four weeks from day of operation the patient was sufficiently recovered to travel and return home (100 miles). She has since fully recovered.

